# Allograft rejection following immune checkpoint inhibitors in solid organ transplant recipients: A safety analysis from a literature review and a pharmacovigilance system

**DOI:** 10.1002/cam4.5394

**Published:** 2022-12-12

**Authors:** Xiangli Cui, Cilin Yan, Ye Xu, Dandan Li, Mingxing Guo, Liying Sun, Zhijun Zhu

**Affiliations:** ^1^ Pharmacy Department of Beijing Friendship Hospital Capital Medical University Beijing China; ^2^ Liver Transplantation Center Beijing Friendship Hospital, Capital Medical University Beijing China; ^3^ School of Automation Science and Electrical Engineering Beihang University Beijing China

**Keywords:** adverse event reporting system, immune checkpoint inhibitors, solid organ transplant, transplant rejection

## Abstract

**Aim:**

This study aimed to systematically characterize transplant rejection after immune checkpoint inhibitors (ICIs) initiation in solid organ transplant recipients (SOTRs).

**Methods:**

Data were extracted from the US FDA Adverse Event Reporting System (FAERS) database and case reports in the literature. Disproportionality analysis including information component and reported odds ratio (ROR) was performed to access potential risk signals.

**Results:**

A total of 168 patients with transplant rejection after ICIs usage were identified in the FAERS database, and 89 cases were identified in the literature review. ICIs were significantly associated with transplant rejection (ROR_025_: 2.2). A strong risk signal was found for combination therapy with pembrolizumab and ipilimumab compared to monotherapy.

**Conclusion:**

Immune checkpoint inhibitors were significantly associated with transplant rejection in SOTRs.

## Graphical abstract

We aimed to systematically characterize transplant rejection after immune checkpoint inhibitors (ICIs) initiation in in solid organ transplant recipients (SOTRs). Data were extracted from the US FDA Adverse Event Reporting System (FAERS) database and case reports in literatures.168 cases in FAERS and 89 cases in literature review were included in this study.

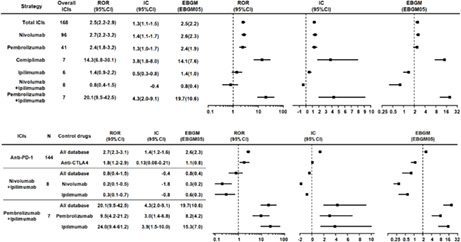

## 1 INTRODUCTION

As most solid organ transplant recipients (SOTRs) require life‐long administration of immunosuppressive agents to maintain allograft tolerance after transplantation, they have an increased risk of primary or recurring tumors, and neoplasm has now become the second most common cause of death among transplant patients.^1^, ^2^ Immune checkpoint inhibitors (ICIs) are considered to be one of the most promising treatments in tumor immunotherapy, and can significantly prolong overall survival time in a variety of cancers, including melanoma, kidney cancer, liver cancer, and lung cancer.^3^, ^4^ Currently, the FDA has approved ICIs including anti‐cytotoxic T‐lymphocyte antigen‐4 (CTLA‐4) monoclonal antibodies (mAbs), such as ipilimumab, anti‐programmed cell death 1 (PD‐1) mAbs, such as nivolumab and pembrolizumab, and anti‐programmed cell death ligand 1 (PD‐L1) mAbs, such as cemiplimab and avelumab.^5^, ^6^ PD‐1 is an ICI expressed on the surface of T cells that is activated mainly by its ligand (PD‐L1), which is expressed or induced in myeloid, lymphoid, and normal epithelial cells. PD‐L1 expression is generally associated with a poorer prognosis and it has been suggested to be a predictive biomarker of the response to anti‐PD‐1/PD‐L1 therapies. CTLA‐4 is a type I transmembrane glycoprotein, a member of the immunoglobulin superfamily that is expressed in both CD4+ and CD8+ T cells.^7^

In SOTRs, immunosuppressants are necessary to prevent graft rejection, and the risk of tumor is increased 2–4 times after SOT.^2^ Although most ICIs instructions do not recommend them for SOT recipients, ICIs are widely used for salvage treatment after tumor recurrence or secondary tumors after SOT, especially when the tumor fails to respond to chemotherapy or targeted therapy. ICIs activate T cells to kill tumors, and the risk of allograft rejection is increased. Immunosuppressants can prevent ICI‐mediated allograft rejection, but they can also compromise the antitumor effect of ICIs.^8^ Thus, how to maintain the balance between immune T‐cell activation and immune suppression in SOTRs is very important and difficult to achieve, and this challenge has attracted the attention of more and more transplantation experts and oncologists.

As most clinical trials of ICIs excluded SOTRs, efficacy and safety data on ICIs in these patients are lacking, and there were only a few reports of ICI‐induced transplant rejection in some single‐center case reports, case series, and systematic reviews.^8^, ^9^, ^10^, ^11^ Data on the safety profile of SOT rejection after administration of various ICIs in post‐marketing studies are scarce. To date, there was only one multi‐center retrospective study on the safety and efficacy of ICIs in 69 cancer patients following kidney transplantation in the past decade, and the results showed that 42% of the patients developed acute rejection after ICI treatment, while only 5.4% developed acute rejection in the non‐ICI cohort.^12^, ^13^ To provide more post‐marketing real‐world data on graft rejection associated with ICIs after organ transplantation for transplantation surgeons using ICIs in SOTRs, we profiled the characteristics of graft rejection associated with ICIs and assessed the risk signals between graft rejection and different ICI regimens. All data are based on the US FDA Adverse Event Reporting System (FAERS) database, and we also summarize the characteristics of transplant rejection after treatment with ICIs in SOTRs from a literature review.

## 2 METHODS

### 2.1 FAERS database analysis

#### 2.1.1 Data source

We performed a retrospective pharmacovigilance study of ICIs‐associated transplantation rejection based on data from January 1, 2011 to June 30, 2021 in the FAERS database (https://open.fda.gov/data/faers/). The FAERS database is a spontaneous reporting system of adverse events filed by health professionals and other non‐healthcare workers. FAERS data files contain demographic and administrative information (DEMO), drug information (DRUG), preferred terms (PTs) coded for the adverse event (REAC), patient outcomes (OUTC), report sources (RPSR), drug therapy start date, event date for reported drugs (THER), and indications for use (INDI).

#### 2.1.2 Drug and adverse event identification

Generic and brand names of ICIs were used to identify transplant rejection reports. MICROMEDEX was used as a dictionary in the generic and brand names for the ICI mining process. Anti‐PD1 agents included nivolumab (Opdivo), pembrolizumab (Keytruda), and cemiplimab (Libtayo). Anti‐PD‐L1 agents included atezolizumab (Tecentriq), avelumab (Bavencio), and durvalumab (Imfinzi). Anti‐CTLA4 agents included ipilimumab (Yervoy) and tremelimumab (Ticilimumab, CP‐675206). ICI monotherapy associated with transplant rejection was notified as “primary suspect” (PS) or “secondary suspect” (SS). Combined therapy is the concurrent usage of any two drugs of PD‐1, PD‐L1, or CTLA‐4 inhibitors as PS, SS or concomitant (C) drugs.

This study included all transplant rejections according to MedDRA version 23.0 (MedDRA ID 23057). In the FAERS database, each report is coded using PTs from MedDRA, the international medical terminology developed by the International Council for Harmonisation of Technical Requirements for Registration of Pharmaceuticals for Human Use. In the FAERS database, all “transplantation rejection” PTs were used to code adverse event reports, including: “Transplant rejection,” “Solid organ transplant rejection,” “Kidney transplant rejection,” “Liver transplant rejection,” “Lung transplant rejection,” “Heart transplant rejection,” “Heart‐lung transplant rejection,” “Intestine transplant rejection,” “Multiple organ transplant rejection,” “Pancreas transplant rejection,” “Liver and pancreas transplant rejection,” “Renal and pancreas transplant rejection,” and “Multiple organ transplant rejection.”

#### 2.1.3 Data mining

Based on the rationale of disproportionality analysis and Bayesian analysis, the reported odds ratio (ROR), the Bayesian confidence propagation neural network (BCPNN), and the multi‐item gamma Poisson shrinker (MGPS) algorithms were employed to investigate the association between different types of ICIs and transplant rejection. We also analyzed the reporting odds ratio (ROR), information component (IC), and empirical Bayesian geometric mean (EGBM) of association between ICIs and transplant rejection in different year reports. The equations and criteria for the three algorithms are listed in Table 1. We also calculated the onset time from administration of ICIs to transplant rejection, which was defined as the interval between the EVENT_DT (adverse event onset date) and the START_DT (start date of the ICI administration). Due to many missing values for START_DT and EVENT_DT, only the transplant rejection onset time of anti‐PD‐1 was counted. The death rates after transplant rejection associated with different ICIs in patients with liver and kidney transplants were compared.

**TABLE 1:** Patient characteristics with ICI‐associated transplant rejection (*n* = 168)

	*n*	%
Reporting region		
America	80	47.6%
Europe	71	42.3%
Asia	0	
Oceania	7	4.2%
Africa	1	0.6%
Missing	9	5.4%
Reporter type		
Healthcare professional	144	85.7%
Non‐healthcare	23	13.7%
Missing	1	0.6%
Reporting year		
2021 Q1–Q2	18	10.7%
2020	36	21.4%
2019	43	25.6%
2018	35	20.8%
2017	27	16.1%
2016	9	5.4%
Gender		
Male	96	57.1%
Female	29	17.3%
Missing	43	25.6%
Age		
Median	64 (14–85)	
<18	4	2.4%
18–44	10	6.0%
45–64	50	29.8%
65–74	50	29.8%
≥75	8	4.8%
Missing	46	27.4%
Anti‐PD‐1	144	85.7%
Nivolumab	96	57.1%
Pembrolizumab	41	24.4%
Cemiplimab	7	4.2%
Anti‐PD‐L1		34.0%
Avelumab	1	0.6%
Anti‐CTLA4		
Ipilimumab	6	3.6%
Combination therapy	17	10.1%
Nivolumab + Ipilimumab	8	4.8%
Pembrolizumab + Ipilimumab	7	4.2%
Pembrolizumab + Nivolumab	2	1.2%
Indications for ICI		
Malignant melanoma	73	43.5%
Lung cancer	28	16.7%
Hepatic cancer	23	13.7%
Skin cancer	15	8.9%
Renal cell carcinoma	5	3.0%
Metastatic carcinoma	3	1.8%
Other carcinoma	9	5.4%
Unspecified	12	7.1%
Type of transplant rejection		
Kidney	104	61.9%
Liver	28	16.7%
Heart	6	3.6%
Lung	1	0.6%
Unspecified	29	17.3%
Time to rejection onset, Median days (min–max)	23 (1–169)	
Kidney	15 (1–85)	
Liver	23 (7–169)	
Outcome		
Hospitalization	25	14.9%
Disability	5	3.0%
Life‐threatening	5	3.0%
Death	54	32.1%

#### 2.1.4 Statistical analysis

We summarized the clinical characteristics of transplant rejection associated with ICIs from the FAERS database and case reports of ICIs‐induced allograft rejection in the literature. ROR was used to calculate the disproportionality, and when the lower end of the 95% confidence interval (CI) of ROR was >1, the signal was considered significant, with more than three cases.^14^ Bayesian confidence propagation neural networks of information components (IC) were used to calculate disproportionality, IC025 > 0 was considered a significant signal.^15^, ^16^ The Pearson's chi‐square test or Fisher's exact test was used to compare the fatality rates between different ICIs, and between liver transplant and kidney transplant patients. Statistical significance was determined at *p* < 0.05 with 95% CIs. We also compared the onset time of transplant rejection using non‐parametric tests, and differences of *p* < 0.05 were considered significant. GraphPad Prism 6.0 (GraphPad Software, California, United States of America) was used for simple comparisons. All the analyses and figure calculations were carried out using Python (version 3.7.0).

### 2.2 Literature review

#### 2.2.1 Search strategy and inclusion criteria

A systematic search of the published literatures was conducted in MEDLINE (from January 2011 to May 2022), EMBASE (from January 2011 to May 2022), and the Cochrane Database of Systematic Reviews (from January 2011 to May 2022). The retrieved references were also reviewed and relevant references were obtained. The abstracts and full texts were reviewed independently by Cui XL and Xu Y, and the search approach is shown in Figure S1. Clinical outcomes were allograft rejection and/or failure after use of ICIs, cancer outcome, and death after ICIs administration. Different decisions were solved by mutual consensus. The included studies fulfilled the following criteria: (i) SOTRs received at least one type of ICIs therapy for malignancy, (ii) SOTRs with an active functioning graft before administration of ICIs. Case reports, case series, and observational studies were included. Conference abstracts, systematic reviews, and review articles were excluded.

#### 2.2.2 Data extraction

We extracted the following information from case reports: family name of the first author, article title, year of publication, sample size, product name and chemical name of ICIs, indication, age, sex, graft function, year after transplant, lag time from ICIs initiation and onset of allograft rejection, pathological features of allograft rejection, follow‐up time after treatment, cancer, and patient outcome (Table 3).

## 3 RESULTS

### 3.1 FAERS database analysis

#### 3.1.1 Descriptive analysis of FAERS database

We screened 8,787,635 reports from the FAERS database and removed duplicated records, according to the FDA's recommendations, by selecting the latest FDA_DT when the CASEID and RE_DT were the same. We finally included 168 reports of ICI‐associated transplant rejection for further analysis (Figure S1), and 168 patients were identified to have SOT rejection events associated with ICIs in the FAERS database from January 1, 2016 to June 30, 2021. The clinical characteristics of these patients are described in Table 1. Most reports were from the America (47.6%) and Europe (42.3%), and the number of cases increased year by year. Healthcare professionals submitted most cases (144, 85.7%). The majority of reported cases were males (96, 57.1%), the median age was 64 years (14–85), and patients older than 65 years accounted for 34.5% of cases. Among the 168 cases of ICI‐associated transplant rejection, kidney transplant rejection and liver transplant rejection accounted for 61.9% and 16.7%, respectively. Nivolumab was associated with the highest number of cases (96, 57.1%), followed by pembrolizumab (41, 2%), and combination therapy with two ICI agents (17, 10.1%) (Figure S2). Malignant melanoma (73, 43.5%), lung cancer (28, 16.7%), and hepatic cancer (23, 13.7%) were the most commonly reported indications for ICIs in SOTRs. The median onset time from initiation of ICI treatment to transplant rejection was 23 (interquartile range [IQR] 1–169) days. We also found that the outcome of transplant rejection was very poor, resulting in 54 deaths (32.1%), 90.74% of which were caused by anti‐PD‐1 agents. The case‐fatality rate appeared to be higher with nivolumab than with pembrolizumab (41.7% vs. 22.0%, *p* = 0.0027) and other ICI regimens. There was no significant difference in the other comparisons.

#### 3.1.2 Concomitant use of ICIs with immunosuppressive agents in the FAERS database

Of 168 cases of transplant rejection associated with the use of anti‐PD1 antibody in the FAERS database, only 66 cases reported using ICIs combined with one to four immunosuppressive agents, most of whom were on a 2‐agent immunosuppressant regimen at the time of ICI treatment (Table S2). Thirty‐six cases used calcineurin inhibitor (CNI)‐containing treatment regimens, 24 cases used mammalian target of rapamycin inhibitor (mTORi) containing regimens, and 48 cases used steroid containing regimens. The most common combination in the immunosuppressive regimen was CNI combined with mycophenolate mofetil (MMF) and steroids. The most common immunosuppressive agents were tacrolimus in combination with MMF and methylprednisolone.

#### 3.1.3 Disproportionality analysis and Bayesian analysis

**FIGURE 1:**
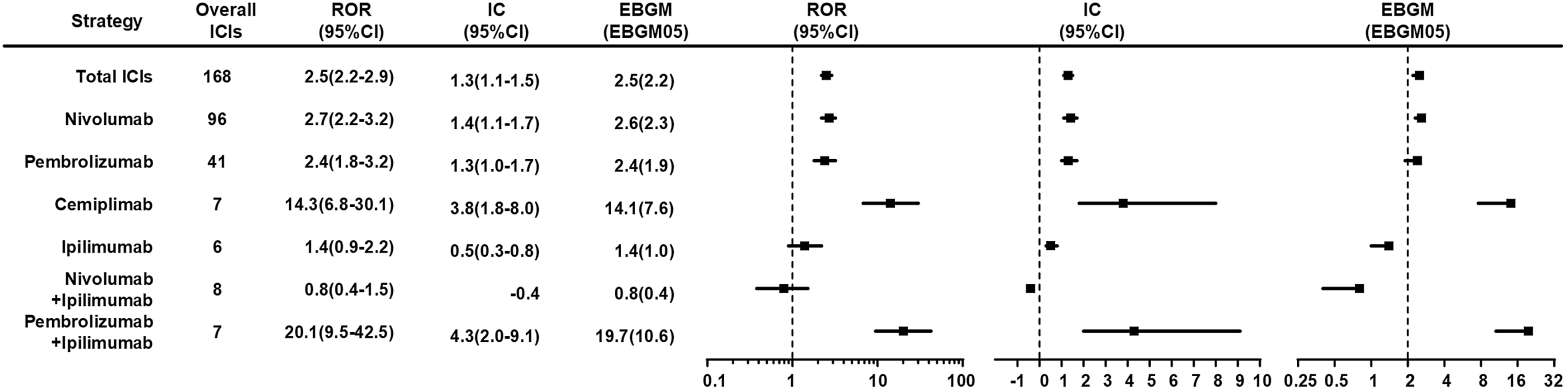
Association of transplant rejection with different types of ICI regimens.

**FIGURE 2:**
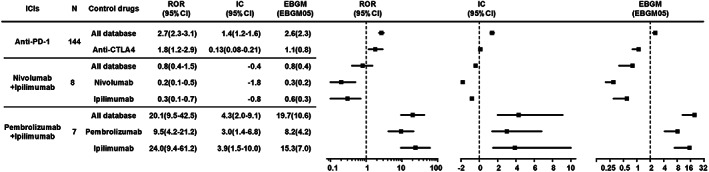
Association of transplant rejection with different types of ICIs (Anti‐PD‐1 vs. anti‐PD‐L1 vs. anti‐CTLA4).

It can be seen in Figure S3 that there were no significant differences in transplant rejection risk signals of ICIs compared with the whole database from 2016 to 2021, which indicated that the deviation caused by possible influencing factors such as policies was small. In general, ICI immunotherapies were significantly associated with over‐reported frequencies of transplant rejection compared with the whole database (Figure 2), corresponding to ROR = 2.5 (2.2–2.9), IC025 = 1.1, and EGBM05 = 2.2. Upon further analysis, a higher frequency of transplant rejection was found for all anti‐PD‐1 agents compared with the whole database, corresponding to ROR = 2.68 (2.3–3.1), IC025 = 1.2, and EGBM05 = 2.3 (Figure 1). Signals were also detected when comparing anti‐PD‐1 agents with anti‐CTLA4 (ipilimumab) (ROR 1.84 [1.2–2.9]) concerning transplant rejection. For combination therapy, pembrolizumab plus ipilimumab also produced a strong signal, corresponding to IC025 = 2.0 and ROR025 = 9.5. In contrast, no signal was detected when nivolumab plus ipilimumab were compared with the whole database, corresponding to IC025 = −0.2 and ROR025 = 0.4 (Figure 4). Anti‐PD‐1 had the risk signals of transplant rejection (ROR 1.84 [1.2–2.9]) when comparing the combination therapy with monotherapy regimens. In addition, signals of transplant rejection were over‐reported for patients treated with combination pembrolizumab plus ipilimumab compared to monotherapy with pembrolizumab (ROR = 9.5, 4.2–21.2) or ipilimumab (ROR 24, 9.4–61.2) (Figure 2).

#### 3.1.4 Time to onset of ICI‐associated transplant rejection in the FAERS database

**FIGURE 3:**
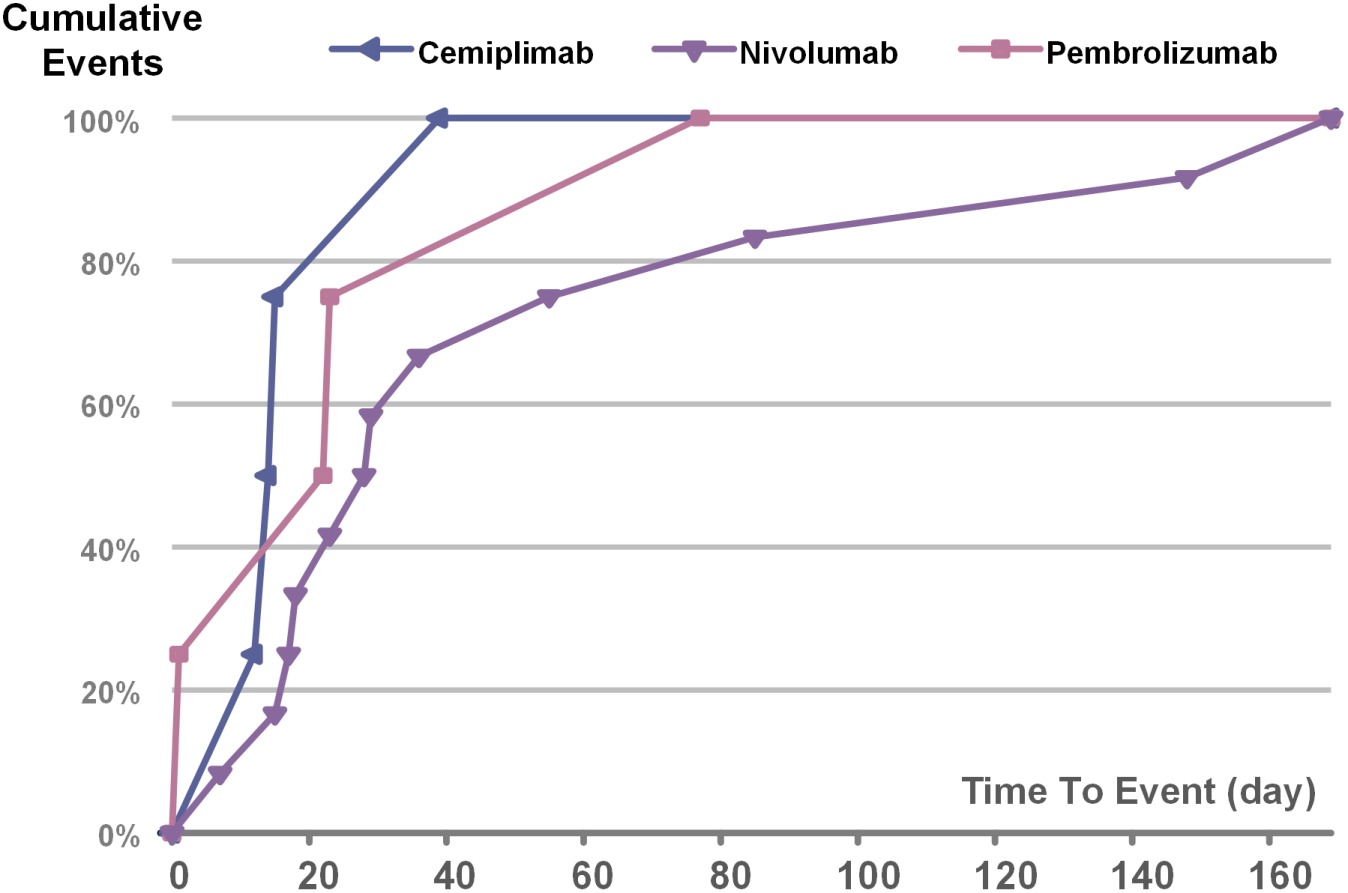
Time (days) to event onset of transplant rejection following different anti‐PD‐1 regimens.

The overall median time from ICI initiation to transplant rejection onset was 23 (interquartile range [IQR]1–169) days, and was similar between liver transplant patients (23, 7–169 days) and kidney transplant patients (21, 1–148 days) (Figure 3). Most transplant rejections (76.2%) occurred within 1–6 weeks after administration of anti‐PD‐1 agents. The median onset times from drug initiation to transplant rejection for nivolumab, pembrolizumab, and cemiplimab were 28.5 (7–169), 23 (1–77), and 14.5 (12–39) days, respectively (Figure S4).

**FIGURE 4:**
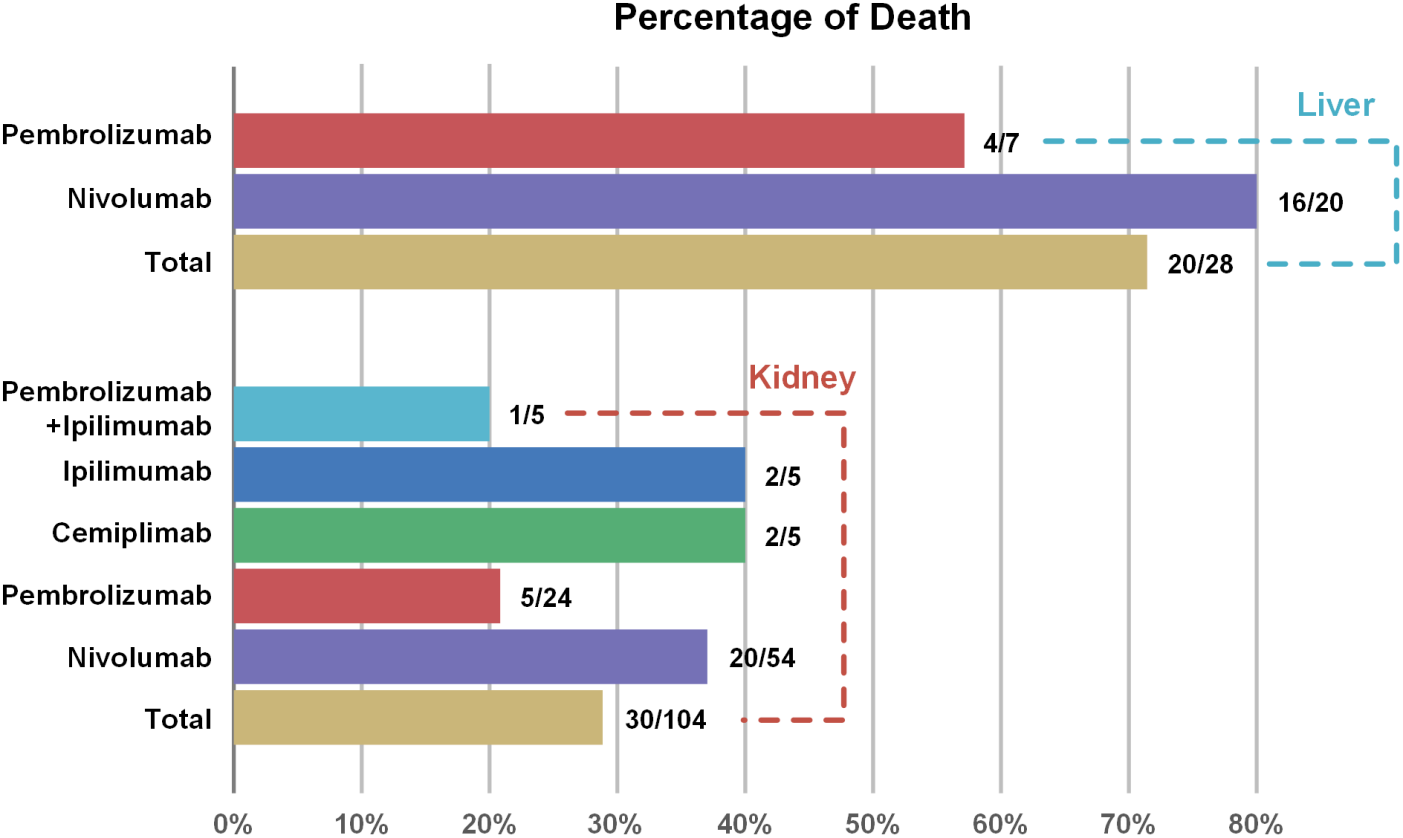
Fatality proportion of liver or kidney transplant rejection following different ICI regimens.

#### 3.1.5 Fatality due to ICI‐associated transplant rejection in the FAERS database

We assessed the mortality rate due to transplant rejection following ICI treatments (Table 2). The total death rate associated with ICIs was 32.1% and anti‐PD‐1 accounted for 96.3% of the total 54 deaths. Among the anti‐PD‐1 monotherapies, nivolumab resulted in the highest fatality rate (42.1%), followed by cemiplimab (40%) and pembrolizumab (22.5%). There was a significant difference in fatality after transplant rejection between nivolumab (40/96, 41.7%) and pembrolizumab (9/41, 22%) (*p* = 0.0027), and fatality after liver transplant rejection (20/28, 71.4%) was higher than that after kidney transplant rejection (30/104, 28.9%) (*p* < 0.0001). Among the six cases of transplant rejection associated with anti‐CTLA4 (ipilimumab), two cases died after transplant rejection. Among the 17 cases treated with dual ICI regimens, only one case (5.9%) died after pembrolizumab plus ipilimumab treatment, and the fatality with monotherapy (53/151, 35.1%) was significantly higher than that with the combination therapy (1/17, 5.9%) with ICIs (*p* = 0.0132).

**TABLE 2:** Fatality proportion of transplant rejection associated with different ICI regimens and different organs of SOT

Overall ICIs	Organ of transplant rejection No		No of death	Percentage
Nivolumab (*n* = 96)	Kidney	54	20	37.74%
	Liver	20	16	80.00%**
	Heart	5	1	20.00%
	Lung	1	1	100.00%
	NA	16	2	12.50%
	Total	96	40	41.67%*
Pembrolizumab (*n* = 41)	Kidney	24	5	21.74%
	Liver	7	4	57.14%**
	Heart	1	0	0.00%
	NA	9	0	0.00%
	Total	41	9	21.95%*
Cemiplimab (*n* = 7)	Kidney	5	2	40%
	NA	2	0	0.00%
	Total	7	2	28.57%
Avelumab (*n* = 1)	Kidney	1	0	0.00%
	Kidney	5	2	40.00%
Ipilimumab (*n* = 6)	Liver	1	0	0.00%
	Total	6	2	33.33%
Nivolumab + Ipilimumab (*n* = 8)	kidney	8	0	0.00%
Pembrolizumab + Ipilimumab (*n* = 7)	Kidney	5	1	20%
	NA	2	0	0.00%
	Total	7	1	14.29%
Pembrolizumab + Nivolumab (*n* = 2)	Kidney	2	0	0.00%
	Total	168	54	32.14%

* *p* < 0.05, the total percentage of death of Nivolumab is higher than that of Pembrolizumab.

** *p* < 0.05, the percentage of death of liver transplant rejection is higher than that of kidney transplant rejection.

### 3.2 Literature review

#### 3.2.1 Characteristics of 89 cases of allograft rejection following ICIs in the literature

Seventy articles including a total of 89 patients who developed allograft rejection after treatment with ICIs were identified (Figure S5, Table 3). The median age was 63 (14–85) years, with 61 males and 38 females. The organs transplanted were as follows: kidney (60 cases), liver (19 cases), heart (5 cases), cornea (3 cases), lung (1 case), and pancreas and kidney (1 case). Most patients had received a PD‐1 inhibitor (38.2%) with nivolumab or pembrolizumab in 34 (38.2%) and 32 (36%) cases, respectively. The predominant indication was melanoma with 37 cases (41.6% of patients). Biopsy‐proven graft rejection was reported in 47 cases [52.8% with acute cellular rejection (29, 61.7%), mixed cellular and antibody‐mediated rejection (15, 16.9%), acute and chronic cellular rejection (2, 2.3%), and antibody‐mediated rejection (1, 1.12%)]. Median time from administration of ICIs to acute rejection diagnosis was 20 (interquartile range, 2–224) days.

**TABLE 3:** Characteristics of 89 cases allograft rejection in SOTRs from literature review

Characteristics	No (%)	Ipilimumab	Nivolumab	Pembrolizumab	Cemiplimab	Toripalimab	Sequential^b^	Combination (Nivolumab + Ipilimumab)
Total	89^a^	6 (6.74%)	34 (38.2%)	32 (35.96%)	6 (6.74%)	1 (1.12%)	5 (5.62%)	4 (4.49%)
Median age (range), years	63 (14–85)	63.5 (40–72)	62.5 (14–84)	63.5 (29–85)	64.5 (51–84)	62 (62)	52 (48–68)	58 (40–72)
Gender								
Male	61 (68.54%)	3	23	21	4		5	4
Female	28 (31.46%)	3	11	11	2	1		
Rejection (allograft failure)								
Kidney	60 (67.42%)	4 (2)	19 (4)	21 (4)	6 (1)		5 (1)	4 (0)
Liver	19 (21.35%)	1 (0)	11 (7)	6 (0)		1 (1)		
Heart	5 (5.62%)		2 (2)	3 (2)				
Cornea	3 (3.37%)	1 (0)	1 (0)	1 (0)				
Lung	1 (1.12%)			1 (1)				
Pancreas + Kidney	1 (1.12%)		1 (0)					
Type of cancer								
MM	37 (41.57%)	5	9	15			5	2
HCC	10 (11.24%)		7	2		1		
cSCC	9 (10.11%)		1	4	3			1
SCC	8 (8.99%)		4	2	2			
RCC	5 (5.62%)		5					
UCC	4 (4.49%)		1	2				1
NSCLC	6 (6.74%)		5	1				
Others	10 (11.24%)	1	2	6	1			
Time from Transplantation to Immunotherapy, median (range), years	10 (0.2–43.2)	15.5 (1.5–19)	5.75 (0.7–26.5)	12.4 (0.2–32)	17 (7–43.2)	1.9 (1.9)	14 (5–15)	5.5 (4–7.8)
Time from Immunotherapy to Rejection, median (range), days	20 (2–224)	20.5 (14–27)	14 (2–168)	45.5 (5–224)	13.5 (4–63)	7 (7)	60 (18–119)	28 (8–28)
Number of doses, median (range)	1.5 (1–14)	1 (1–4)	1 (1–14)	2 (1–11)	2.5 (1–7)	1 (1)	3 (2–5)	1 (1–2)
Immunesuppression at time of initiation of immunotherapy								
Single agent	40 (44.94%)							
Steroids	25 (28.09%)	4	4	10	3		4	
Sirolimus	5 (5.62%)	1	3	1				
CSA	2 (3.37%)			2				
MMF	2 (2.25%)		2					
Everolimus	2 (2.25%)		1					
Tacrolimus	3 (3.37%)		2	1				
Sirolimus or Everolimus	1 (1.12%)					1		
Combination	32 (35.96%)							
2 drugs combination	25 (28.09%)	1	10	10	2		1	1
3 frugs combination	6 (6.74%)		3	2	1			
4 drugs combination	1 (1.12%)			1				
Tacrolimus alone or in combination	19 (21.35%)		10	6	2		1	
Steroids alone or in combination	45 (49.44%)	5	12	17	6		4	1
No Immunesuppression	5 (5.62%)		2	2				1
N/A	12 (13.48%)		7	3				2
Immune checkpoint expression								
Graft positive	7 (7.87%)		4	2		1		
Graft negative	1 (1.12%)		1					
Tumor positive	1 (1.12%)		1					
Tumor negative	1 (1.12%)		1					
Graft + Tumor positive	2 (2.25%)		1	1				
N/A	77 (86.52%)	6	26	29	6		5	4
Prior history of significant rejection								
Yes	5 (5.62%)	1	4					
No	62 (69.66%)	4	26	19	3	1	5	3
N/A	22 (24.72%)	1	4	13	3			1
Type of Rejection (biopsy)								
Acute cellular rejection	29 (32.58%)	3	11	8	2		3	1
Mixed acute cellular and antibody mediated rejection	13 (14.61%)		6	3	1		1	2
Acute and chronic cellular rejection	2 (2.25%)			2				
Acute cellular rejection (5 days) Antibody mediated rejection (7 m)	1 (1.12%)			1				
Acute cellular rejection (32 days) Antibody mediated rejection (100 days)	1 (1.12%)		1					
Acute antibody mediated rejection	1 (1.12%)			1				
No Biopsy	10 (11.24%)	1	5	2	2			
N/A	32 (35.96%)	2	11	15	1	1	1	1
Response to therapy								
Response rate (CR + PR)	23 (25.84%)	0	8	10	0	0	3	2
CR	9 (10.11%)		2	5			1	1
PR	14 (15.73%)		6	5			2	1
SD	6 (6.74%)		1	2	2			1
PD	31 (34.83%)	5	13	8	3		1	
N/A	29 (32.58%)	1	12	12	1	1	1	1
Outcome								
Live	33 (37.08%)	1	8	17	2		3	2
Death	40 (44.94%)	5	19	10	3	1		1
Allograft failure	26 (29.21%)	2	13	7	1	1	1	
N/A	16 (17.98%)		7	5	1		2	1

Abbreviations: AZA, azathioprine; CNI, calcineurin inhibitor; CSA, ciclosporin A; CR, complete response; MM, malignant melanoma; HCC, hepatocellular carcinoma; cSCC, cutaneous squamous cell carcinomas; NSCLC, non‐small cell lung cancer; SCC, squamous cell carcinomas; RCC, renal cell carcinoma; UCC, urothelial cell cancer; MMF, mycophenolate mofetil; mTOR inhibitors, mammalian target of rapamycin inhibitor; PD, progressive disease; PR, partial response; SD, stable disease.

^a^ One patient with an unclear drug, anti‐pd‐1, kidney failure.

^b^ Pembrolizumab followed by nivolumab (1), ipilimumab followed by pembrolizumab (3), Ipilimumab followed by nivolumab (1).

#### 3.2.2 Patient outcomes

Overall, of the 89 SOTRs who developed graft rejection, survival data were reported in 73 patients. Thirty‐three cases (37.1%) were reported to be alive at the end of follow‐up. Forty cases (44.9%) died of various etiologies, 21 (21/60, 35%) cases of kidney transplant, 14 (14/19, 73.7%) cases of liver transplant and 5 (5/5,100%) cases of heart transplant. Eighteen cases (45%) died of allograft failure after graft rejection, and other deaths were related to cancer progression. Overall, the details of cancer outcome were reported in 60 patients. Complete response, partial response and stable disease were noted in 9 (10.1%), 14 (15.7%), and 6 (6.7%) patients, respectively. Progressive disease was reported in 31 (34.8%) patients.

## 4 DISCUSSION

ICIs including PD‐1, PD‐L1, and CTLA‐4 inhibitors have shown survival benefit in multiple malignancies,^1^, ^2^, ^3^ but their immune‐related adverse events have been widely reported in many systems.^4^ SOTRs have a high risk of new occurrence and recurrence of neoplasm due to immunosuppressive treatments or oncogenic viral infections, and neoplasm has now become the second most common cause of death among transplant patients.^4^ Lipson EJ first reported the successful use of ICIs in SOTRs with anti‐CTA4 ipilimumab used in two kidney transplantation patients with metastatic melanoma in 2014.^20^ However, during the past 8 years, efficacy and safety data for ICIs in patients who have undergone SOT are lacking as they were excluded from most clinical trials of ICIs for malignancies. Studies have suggested that approximately 40% of SOTRs treated with ICIs are likely to experience transplant rejection.^9^, ^17^, ^18^, ^19^, ^20^ Regardless of the cancer type, the median overall survival (5 months) was lower in SOT patients who suffered graft rejection than in those who did not have graft rejection.^21^ Due to the high risk of transplant rejection after ICIs usage, transplant surgeons and oncologists are very concerned about the safety of ICIs treatment before and after transplantation in SOTRs, and whether it is necessary to permanently discontinue ICIs in SOT after allograft rejection. However, data are limited to a few single‐center case series and case reports.^6^, ^7^, ^8^

We examined the profile and risk signals of transplant rejection associated with ICI therapy, and compared the fatality rates of different ICI regimens and different organs after transplant rejection using data from the FAERS database (168 cases) and case reports in the literature (89 cases). In our study, a higher rate of transplant rejection was observed with all anti‐PD‐1 agents (144/168, 85.7%) (nivolumab 57.1%, pembrolizumab 24.4%, cemiplimab 4.2%) and combination therapy with pembrolizumab and ipilimumab compared with the whole FAERS database. In addition, in the 89 cases reported in the literature, the predominant agents were anti‐PD‐1 agents in 72 cases (80.9%) (nivolumab 38.2%, pembrolizumab 36%, cemiplimab 6.7%) which also induced allograft rejection. Only one multi‐center retrospective study analyzed the safety and efficacy of ICIs in 69 cancer patients who underwent kidney transplantation over the past 10 years, and the results showed that 42% (29/69) of patients developed acute rejection after ICI treatment, whereas only 5.4% developed acute rejection in the non‐ICI cohort.^12^ Anti‐PD‐1 therapies have the highest risk of graft rejection (44%, 22/50), and account for 75.9% of total graft rejections, the other 20.7% rejections were due to PD‐1/CTLA‐4 combination.^22^, ^23^ The detection of PD‐L1 expression in biopsy specimens may be useful for identifying the potential transplant rejection population following ICIs. In our study, PD‐L1 was detected in 12 of 89 cases, 7 positive in graft, 1 positive in tumor and 1 in both graft and tumor.

In our study of the FAERS, the risk signals were not found in anti‐CTLA4 agents, and only six cases of transplant rejection occurred in SOTRs treated with ipilimumab, which was the first ICI used for melanoma in 2011. In addition, six cases of transplant rejection occurred in 89 case reports when treated with ipilimumab. From the limited cases reported, there was some suggestion that SOTRs treated with CTLA‐4 inhibitors are less likely to experience rejection and graft loss.^21^, ^24^, ^25^ Some experimental studies investigated the roles of the immune regulatory pathway in graft tolerance, and the PD‐1/PD‐L1 pathway seems to be more important for the maintenance of graft acceptance, whereas the CTLA‐4 pathway may be primarily involved in graft tolerance induction.^26^, ^27^, ^28^

Death primarily from allograft rejection or rejection complications was reported in approximately 40–50% of patients in the literature review or case series.^19^, ^20^, ^23^ In our study, which included 168 cases of graft rejection in SOTRs treated with ICIs in the FAERS database, the total death rate was 32.1% (54/168), and death after graft rejection in liver recipients was higher than that in kidney recipients (71.4% vs. 28.9%). In the 89 case reports, the total death rate was 44.9% in the transplant rejection population, with 73.7% in liver transplant patients, 35% in kidney transplant patients, and 100% in heart transplant patients, which was consistent with the results of the FAERS and systematic reviews. One systematic review included 83 SOTRs treated with ICIs and reported that the death rate was 66.7% (16/24), 52.8% (28/53), and 66.7% (4/6) in liver recipients, kidney recipients, and heart recipients, respectively.^19^ Another patient‐centered systematic review included 57 SOTRs (kidney 32, liver 20, heart 5) treated with ICIs for various metastatic cancers, and death secondary to graft rejection was 85.7% (6/7) and 15.4% (2/13) in liver recipients and kidney recipients, respectively.^13^ One case series included 14 liver transplant recipients treated with ICIs, and 75% (3/4) died after acute transplant rejection within 3 weeks after initiation of nivolumab or pembrolizumab therapy.^29^ Lethal outcomes were common after liver transplant rejection.^30^ Our study and most previous systematic reviews found that liver allograft rejection and heart allograft rejection resulted in higher death rates than kidney transplant rejection. Dialysis can be used for kidney transplant recipients after graft rejection with ICIs and perhaps explains the lower fatality rate in these patients compared to that in liver or heart transplant patients after allograft rejection.^22^

In the present study, we found that the death rate associated with nivolumab was significantly higher than that for pembrolizumab and other ICI agents after transplant rejection. However, the difference between anti‐PD‐1 agents, anti‐CTLA4 agents, and combination therapy was not significant in the 168 cases from the FAERS database. The death rate was also higher than in our study of 89 case reports, the death rate was 58.8% and 31.2% for nivolumab and pembrolizumab, respectively, after transplant rejection induced by ICIs. In one systematic review, the death rate after transplant rejection was higher after nivolumab (75%, 3/4) treatment than that after pembrolizumab treatment (41.7%, 5/12).^23^ Due to the limited amount of data and different causes of death, we are unable to draw a conclusion as to which type of ICI had a higher risk of death, and more multi‐center prospective studies are needed to definitively answer this question.

In the FAERS database, we found the median time to onset of graft rejection associated with ICIs was 23 days, and most cases (76.2%) appeared within the first six weeks after ICI initiation, which was similar to the results of the literature review, where the median time from onset of ICIs to acute rejection diagnosis was 20 days. These results were similar to one study in which the median time to onset of graft rejection associated with ICIs was 21 days (13–56).^24^ There was no difference between the ICIs administered. A phase I study showed that the serum half‐life of anti‐PD‐1 was 12–20 days. However, pharmacodynamics indicated a sustained 2 months of more than 70% occupancy of PD‐1 on T cells following various infusion doses.^25^ Therefore, when to stop ICIs before organ transplantation is still a problem to be solved for all transplant surgeons and scientists. More in‐depth basic experimental research data should be carried out to support the decision‐making basis for clinical provision of safe treatment before ICIs are administered to SOT patients.

In our study, most transplant biopsies demonstrated an acute T lymphocyte‐mediated rejection (61.7%, 29/47) process in patients who received ICIs. Mixed cellular and antibody mediated rejection (27.7%, 13/47) was also observed in other patients. These results are consistent with a study which showed that acute cellular rejection was found in 61.1% of cases and mixed cellular and antibody‐mediated rejection was found in the others.^19^ Positive PD‐L1 was found in 9 of 89 case reports (only 12 detected). Most reported transplant rejections after ICI treatment were T‐cell‐mediated, and only a small proportion showed antibody‐mediated rejection.^26^, ^27^, ^28^ The main reason for this is that anti‐PD‐1, anti‐PD‐L1, and anti‐CTLA4 ICIs can activate T cells, and induce graft rejection. Some studies recommend staining for PD‐L1 using the pretherapeutic biopsy from the graft before anti‐PD‐1 mAb administration, as PD‐L1 positive staining may predict graft rejection, whereas negative PD‐L1 staining indicates that anti‐PD‐1 can be used safely.^29^, ^30^, ^31^ Pretreatment with steroids and changes in the immunosuppressive regimens are potential strategies that should be considered to prevent transplant rejection when administering ICIs to SOTRs.^32^ Although ICIs have a powerful effect in cancer immunotherapy, they should be used with extreme caution in SOTRs with malignancies.

In this study, steroids, CNIs, and mTORi were the most popular ICIs used in SOTRs in the FAERS database and case reports. After rejection, immunosuppressive agents were adjusted in most cases, by a reduction in dosage or types, and withdrawal of CNI and a change to mTORi. Single agent or 2 agents in combination were demonstrated to be popular in our results. Immunosuppressive agents are a double‐edged sword. On the one hand, they can inhibit T‐cell growth or differentiation, in order to protect the function of the allograft and prevent transplant rejection. On the other hand, they can increase the risk of tumor occurrence and recurrence. The potential confounding factor of transplant rejection is that immunosuppressive drugs are often reduced or discontinued, thus increasing the risk of transplant rejection.^33^ mTORi has been shown in animal models and clinical trials to inhibit tumor growth and progression, and is considered to lower the risk of graft rejection.^34^

To the best of our knowledge, we report the largest and most extensive pharmacovigilance study on transplant rejection following ICI treatment, with 168 cases derived from the FAERS database and 89 case reports from the literature. The transplant rejection signals of these ICIs are robust, which indicate that possible influencing factors such as policy have resulted in small deviations from 2016 to 2021 (Figure S3). We identified some of the characteristics of transplant rejection induced by ICIs in a real‐world study from the FAERS database and case reports, and these data can provide some reference for clinicians before using ICIs in SOT patients. They should consider the risk of transplant rejection, the appropriate time to administer ICIs, and weigh the pros and cons of tumor progression and transplant rejection.

There are some limitations in our study. First, FAERS is a voluntary reporting system, and it did not cover all the cases where ICI‐associated transplant rejection occurred in the real world, and the incidence rate of transplant rejection and fatality rate could not be calculated due to the lack of the accurate number of SOTRs treated with ICIs. Similarly, we were unable to present the efficacy of ICIs as survival analysis and prognosis given the nature of the data available in the FAERS database. Second, the data format in the FAERS database was not standardized as in a RCT or cohort clinical trials, which may lead to bias in our results. Third, the accurate transplant operation time was not available in the FAERS database. Therefore, we could not judge the order of ICI administration and SOT, and missing data of transplant rejection time >5% in this study. Although the FAERS database has some inherent limitations, it characterizes the risks of transplant rejection associated with different types of ICIs, and can provide some guidance for the use of ICIs in SOTRs. In order to make up for the limitations in FAERS, we also analyzed case reports to identify the characteristics of the allograft after SOT, and most results were consistent with the data in the FAERS database.

## 5 CONCLUSIONS

SOTRs have a risk of allograft rejection following ICI initiation, and a possible higher death rate after transplant rejection, especially the liver or heart transplant recipients, compared with kidney transplant recipients. Clinicians should evaluate the risk/benefit ratio for SOTRs with tumors before using ICIs. Further prospective studies should be conducted to investigate the effects of ICI agents in SOTRs, in order to help clinicians delineate a subset of SOTRs who can benefit from ICI treatment. PD‐L1 positive expression in graft biopsy may be an effective marker for predicting transplant rejection. A national registry of SOTRs treated with ICIs should be considered, and SOTRs undergoing ICIs could be enrolled in multi‐center post‐marketing phase IV clinical trials.

## AUTHOR CONTRIBUTIONS

**Xiangli Cui:** Conceptualization (lead); project administration (supporting); writing – original draft (lead). **Cilin Yan:** Data curation (equal); methodology (supporting); software (supporting); visualization (equal). **Ye Xu:** Methodology (supporting); supervision (supporting); visualization (supporting). **Dandan Li:** Investigation (supporting); resources (supporting). **Mingxing Guo:** Data curation (supporting); resources (supporting); supervision (equal). **Liying Sun:** Project administration (lead); supervision (supporting). **Zhijun Zhu:** Investigation (lead); project administration (lead); supervision (lead).

## FUNDING INFORMATION

National natural science foundation of China (81970562).

## CONFLICT OF INTEREST

All authors declare that they have no conflict of interest.

## Supporting information


Table S1
Table S2

Figure S1

Figure S2

Figure S3

Figure S4

Figure S5

## Data Availability

All data are available within the manuscript and supplemental materials, and FAERS database was available at website (showed in the data source): https://open.fda.gov/data/faers/.
