# Supplementary material for: Allograft rejection following immune checkpoint inhibitors in solid organ transplant recipients: A safety analysis from a literature review and a pharmacovigilance system
Source: Cancer Med. 2022 Dec 12;12(5):5181–94. doi: 10.1002/cam4.5394 (PMC10028127; doi:10.1002/cam4.5394)
Supplement: Supplementary file 1 — Table S1 Table S2 [file CAM4-12-5181-s004.docx]

**Allograft rejection following Immune Checkpoint Inhibitors in solid organ transplant recipients: a safety analysis from literature review and a pharmacovigilance system**

**Appendix:**

**Abbreviations:**

FAERS- US Food and Drug Administration (FDA) Adverse Event Reporting System MedDRA-Medical Dictionary for Regulatory Activities

SOT-solid organ transplantation

SOTRs-solid organ transplant recipients

ICI-immune checkpoint inhibitor

AE-adverse event

BCPNN- Bayesian confidence propagation neural network

EBGM-empirical Bayesian geometric mean

EBGM05-the lower 95% one-sided CI of EBGM

PD-L1-programmed death-ligand 1

PD-1- programmed cell death protein 1

CTLA-4-cytotoxic T lymphocyte-associated protein 4

FAERS-US FDA Adverse Event Reporting System

PRR-proportional reporting ratio

RORs-reporting odds ratios

CI-confidence intervals

IC-information component

IC025-the lower limit of the 95% two-sided CI of the IC

IQR-information component; interquartile range

MGPS-multi-item gamma Poisson shrinker; N, the number of co-occurrences

PS- primary suspected

SS-secondary suspected

PT- preferred term;

CNI-Calcineurin Inhibitor

MMF-Mycophenolate Mofetil

irAEs- immune-related adverse events

mTORi-Mammalian target of rapamycin inhibitors

mAbs-monoclonal antibodies

χ2- chi-squared

**Supplemental Tables**

Table S1. Summary of major algorithms used for signal detection

| Algorithms | Equation* | Criteria |
| --- | --- | --- |
| $\text{ROR}$ |  | $\begin{matrix} 95\%\text{CI}>1, \\ \text{N}\geq2 \end{matrix}$ |
| $\text{PRR}$ | $\begin{matrix} \text{PRR}=(\text{a}/(\text{a}+\text{c}))/(\text{b}/(\text{b}+\text{d})) \\ \chi^{2}=\Sigma((\text{O}-\text{E})^{2}/\text{E}) \\ (\text{O}=\text{a},\text{E}=(\text{a}+\text{b})(\text{a}+\text{c})/(\text{a}+\text{b}+\text{c}+\text{d})) \end{matrix}$ | $\begin{matrix} \text{PRR}\geq2, \\ \chi^{2}\geq4,\text{N}\geq3 \end{matrix}$ |
| $\text{BCPNN}$ |  | $\begin{matrix} \text{IC>0,} \\ \text{IC025>0} \end{matrix}$ |
| $\text{MGPS}$ |  | $\begin{matrix} \text{EBGM05>2,} \\ \text{ N>0} \end{matrix}$ |

**Abbreviations:** BCPNN, Bayesian confidence propagation neural network; CI, confidence interval; EBGM, empirical Bayesian geometric mean; EBGM05, the lower 90% one-sided CI of EBGM; IC, information component; IC025, the lower limit of the 95% two-sided CI of the IC; MGPS, multi-item gamma Poisson shrinker; N, the number of co-occurrences; PRR, proportional reporting ratio; ROR, reporting odds ratio; χ2, chi-squared. *a: number of reports containing both the suspect drug and the suspect adverse drug reaction. b: number of reports containing the suspect adverse drug reaction with other medications (except the drug of interest). c: number of reports containing the suspect drug with other adverse drug reactions (except the event of interest). d: number of reports containing other medications and other adverse drug reactions.

Table S2. Immunosuppression types

| **Kinds** | **Drugs** | **N(%)** |
| --- | --- | --- |
| Single agent  (n=20) | CNI | 20(30.3)  4 |
|  | MMF | 8 |
|  | Steroid | 8 |
| 2 drugs  (n=26) | CNI+MMF | 26(39.4)  5 |
|  | CNI+Steroid | 7 |
|  | CNI+mTORi | 3 |
|  | mTORi+MMF | 3 |
|  | MMF+Steroid | 1 |
|  | MMF+mTORi | 4 |
|  | Antimetabolite+mTORi | 2 |
|  | Antimetabolite+Steroid | 1 |
| 3 drugs  (n=16) | CNI+MMF+Steroid | 16(24.2)  9 |
|  | CN+MMF+mTORi | 3 |
|  | MMF+Steroid+mTORi | 2 |
|  | CNI+mTORi+Steroid | 2 |
| 4 drugs  (n=4) | CNI+mTORi+MMF+Steroid | 4(6)  4 |
|  | **Total** | 66 |
