# Supplementary figures and images for: Allograft rejection following immune checkpoint inhibitors in solid organ transplant recipients: A safety analysis from a literature review and a pharmacovigilance system
Source: Cancer Med. 2022 Dec 12;12(5):5181–94. doi: 10.1002/cam4.5394 (PMC10028127; doi:10.1002/cam4.5394)

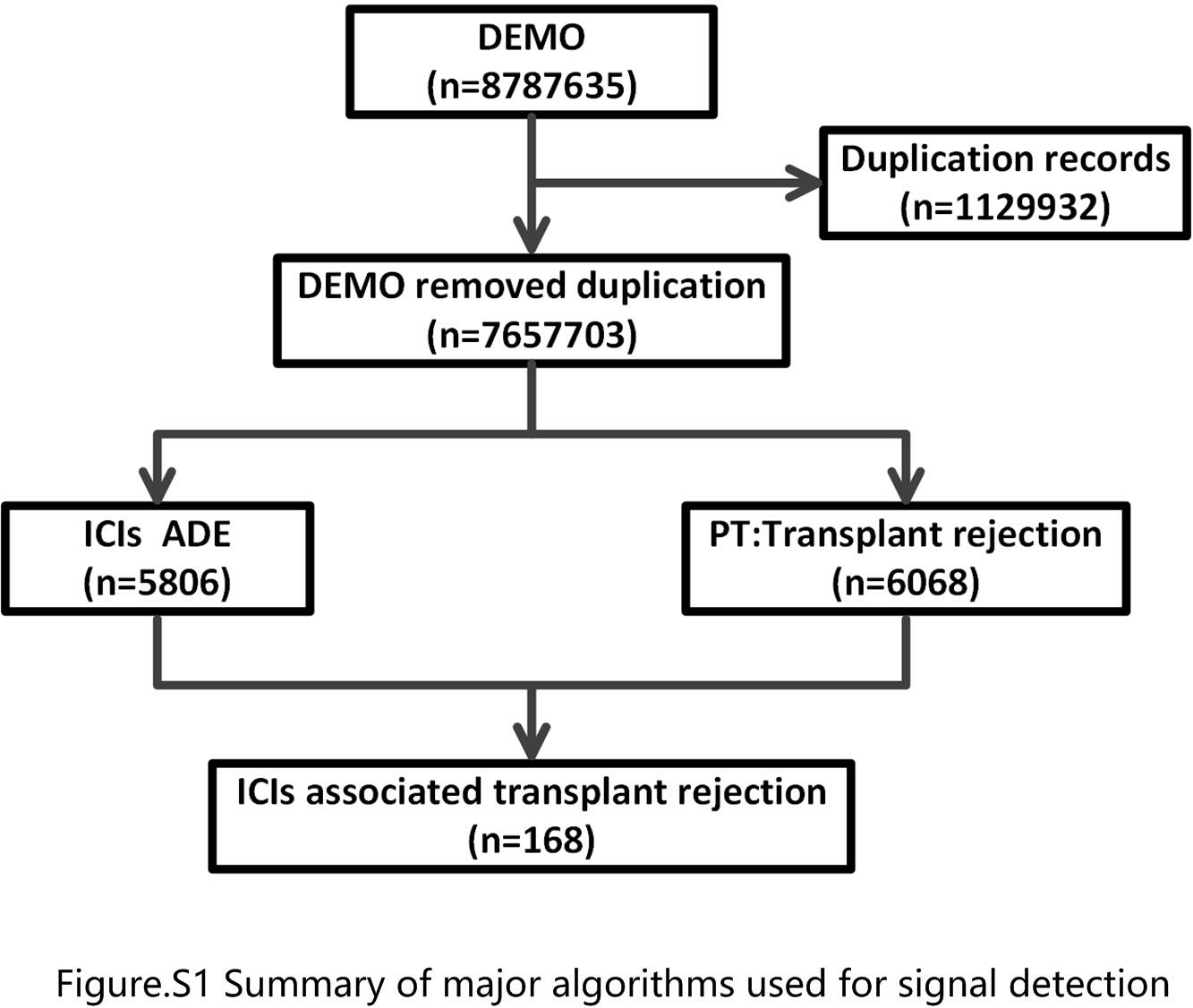

Supplement: Supplementary file 2 — Figure S1 [file CAM4-12-5181-s003.tif]

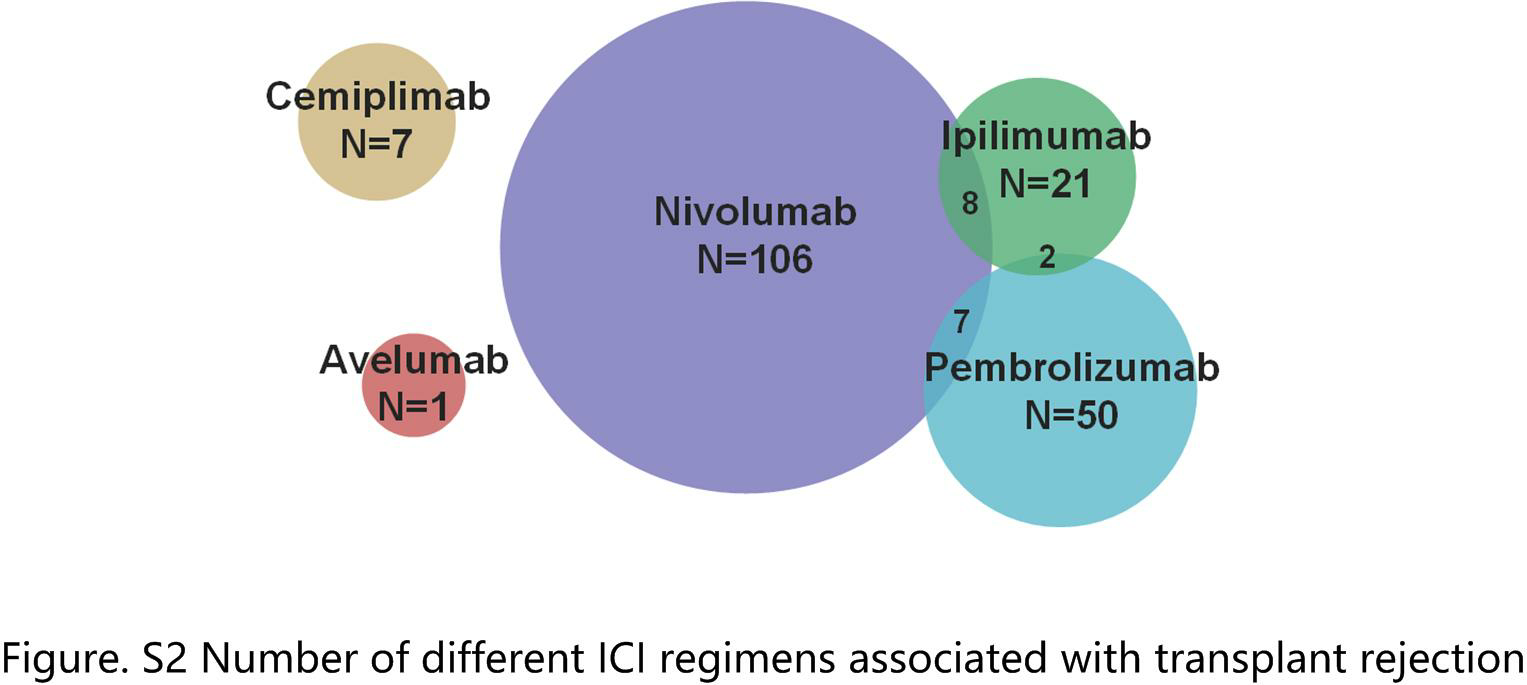

Supplement: Supplementary file 3 — Figure S2 [file CAM4-12-5181-s002.tif]

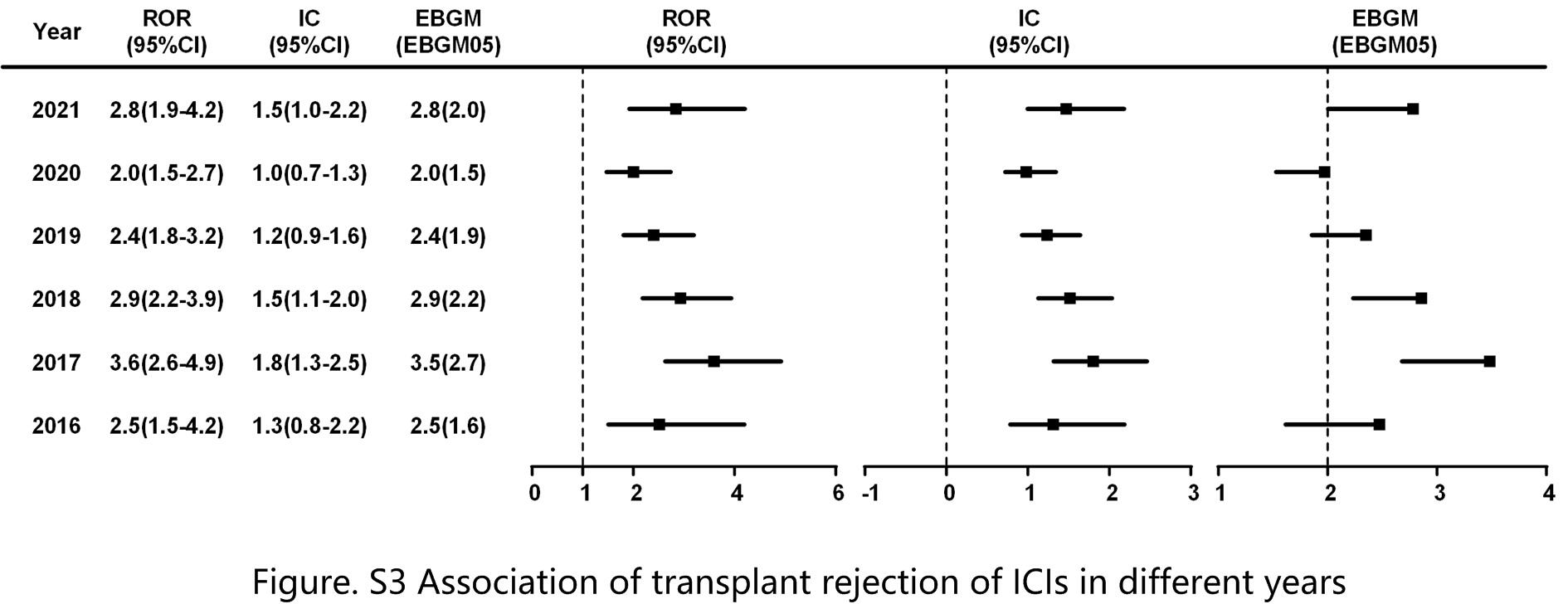

Supplement: Supplementary file 4 — Figure S3 [file CAM4-12-5181-s006.tif]

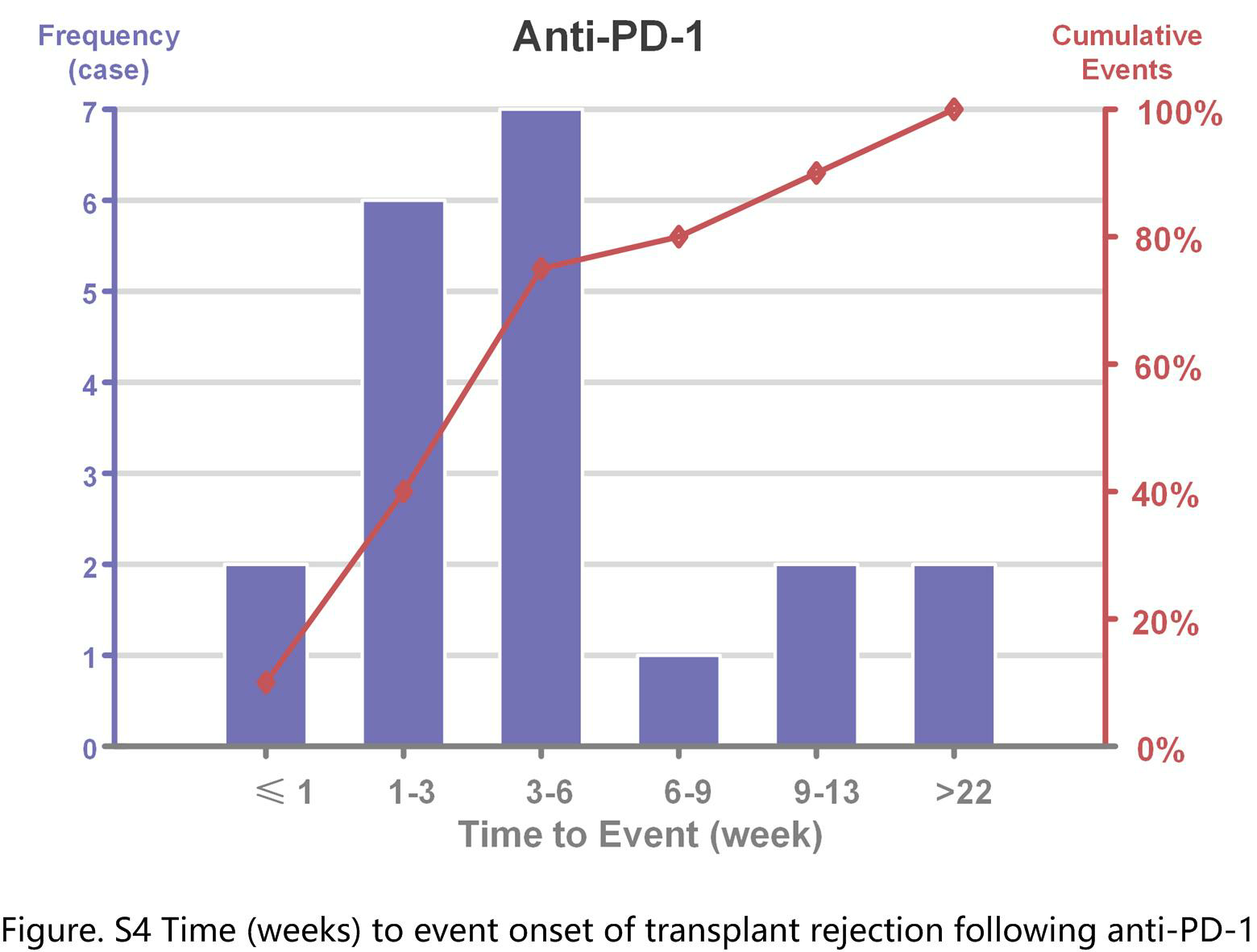

Supplement: Supplementary file 5 — Figure S4 [file CAM4-12-5181-s005.tif]

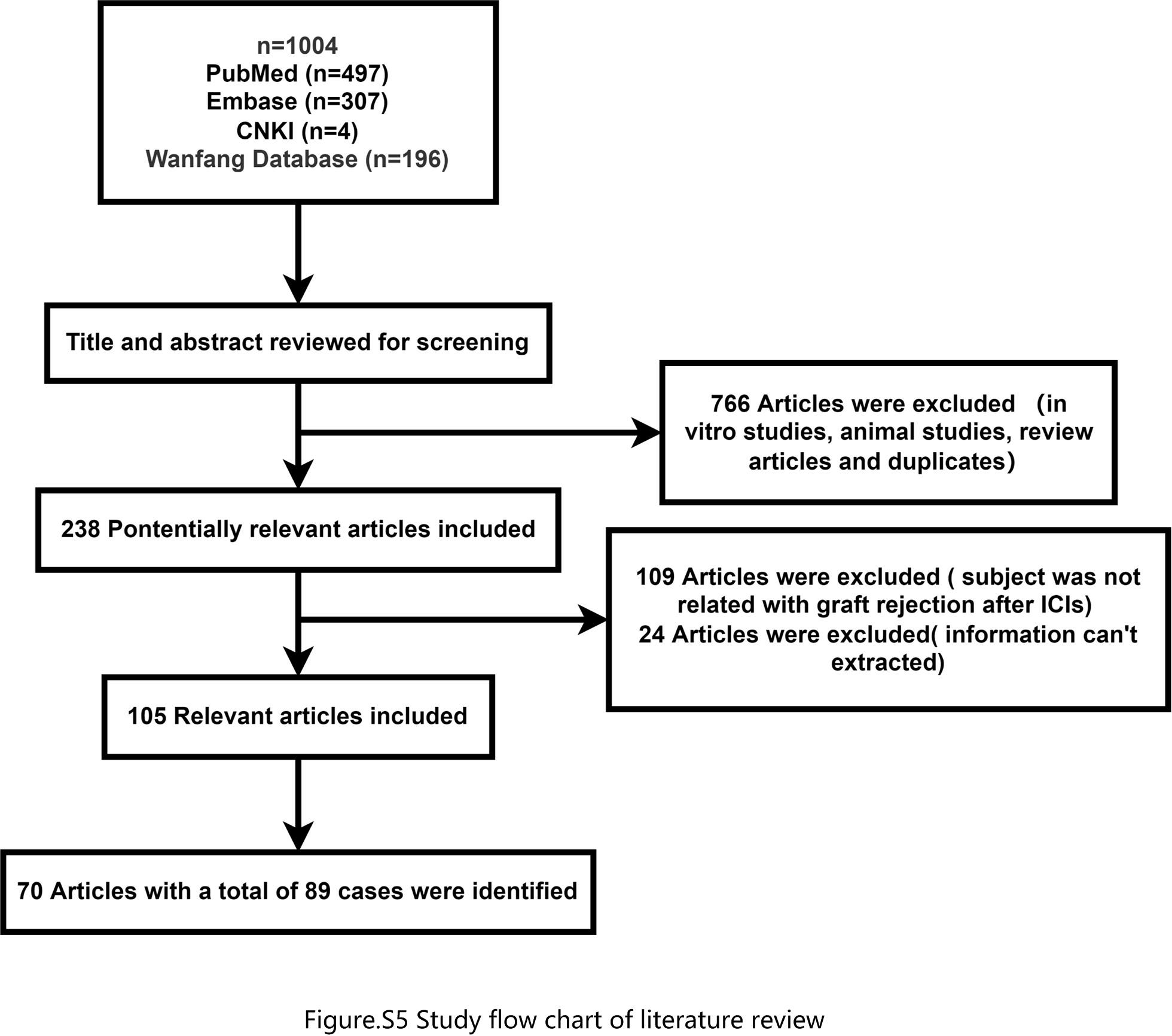

Supplement: Supplementary file 6 — Figure S5 [file CAM4-12-5181-s001.tif]
